# Signal Denoising Method Using AIC–SVD and Its Application to Micro-Vibration in Reaction Wheels

**DOI:** 10.3390/s19225032

**Published:** 2019-11-18

**Authors:** Xianbo Yin, Yang Xu, Xiaowei Sheng, Yan Shen

**Affiliations:** College of Mechanical Engineering, Donghua University, Shanghai 201620, China; yinxb_2008@163.com (X.Y.); shengxw@dhu.edu.cn (X.S.); shenyan1871@126.com (Y.S.)

**Keywords:** signal denoising, singular value decomposition, Akaike information criterion, reaction wheel, micro-vibration

## Abstract

To suppress noise in signals, a denoising method called AIC–SVD is proposed on the basis of the singular value decomposition (SVD) and the Akaike information criterion (AIC). First, the Hankel matrix is chosen as the trajectory matrix of the signals, and its optimal number of rows and columns is selected according to the maximum energy of the singular values. On the basis of the improved AIC, the valid order of the optimal matrix is determined for the vibration signals mixed with Gaussian white noise and colored noise. Subsequently, the denoised signals are reconstructed by inverse operation of SVD and the averaging method. To verify the effectiveness of AIC–SVD, it is compared with wavelet threshold denoising (WTD) and empirical mode decomposition with Savitzky–Golay filter (EMD–SG). Furthermore, a comprehensive indicator of denoising (CID) is introduced to describe the denoising performance. The results show that the denoising effect of AIC–SVD is significantly better than those of WTD and EMD–SG. On applying AIC–SVD to the micro-vibration signals of reaction wheels, the weak harmonic parameters can be successfully extracted during pre-processing. The proposed method is self-adaptable and robust while avoiding the occurrence of over-denoising.

## 1. Introduction

As a most common mechanical device, rotating machinery plays a vital role in modern industry. Unlike general equipment, rotating machinery is typically operated in harsh, high-speed, and heavy-load environments. These conditions can easily harm the key components of a mechanical system, such as gears, bearings, and rotors. With further expansion, the damage can cause equipment failure and even casualties. To ensure the safe operation of rotating machinery, fault detection techniques including vibration analysis, acoustic emission, temperature analysis, and wear debris analysis have been developed [1]. Among them, vibration analysis is widely used, owing to its signal testability and high correlation with structural dynamics. Simultaneously, in the fault diagnosis of rotating machinery, the corresponding signal processing technologies have been a part of the most useful approaches [2].

Considering the environmental and structural factors, the source signals are commonly mixed with random noise, which is problematic for the early fault detection of machinery [3]. For the purpose of extracting effective information, numerous reasonable methods are applied to reduce the noise from measured vibration signals. Affected by a series of non-linear factors, such as internal friction, loads, stiffness, and assembly gap, the vibration signals of rotating machinery have strong non-linear and non-stationary characteristics [4]. As powerful tools for non-stationary signal processing, time–frequency analysis methods are commonly used to analyze the characteristics of vibration signals. In general, time–frequency analysis methods include short-time Fourier transform (STFT), discrete wavelet transform (DWT), empirical mode decomposition (EMD) [5], local mean decomposition (LMD) [6], and variational mode decomposition (VMD) [7]. Concurrently, in practical applications, the denoising methods based on time–frequency analysis have also made significant contributions. Currently, the methods based on wavelet analysis are the most well-known processing methods of signal denoising [8]. As a typical approach, based on the multi-resolution and self-similar characteristics of wavelet analysis, wavelet threshold denoising (WTD) reduces the noise in non-stationary signals [9]. In engineering applications, there are still a few limitations in WTD, such as the selection of the wavelet basis functions [10] and phase lag after denoising [11]. Similar to WTD, the quality of EMD threshold denoising strongly depends on the selection of threshold parameters [12]. To achieve ideal denoising, comparatively more advanced denoising methods are developed by the improvement of time–frequency analysis, such as EMD with Savitzky–Golay filter (EMD–SG) [13]. Apart from time–frequency analysis, significant research efforts have been made for realizing noise reduction, such as singular value decomposition (SVD) [14], matching tracking [15], and sparse representation [16].

SVD is a non-parametric technique first proposed by Beltrami in 1873 [17]. In engineering applications, signal processing based on SVD has been an effective approach to analyze non-linear and non-stationary signals. It has been utilized in various applications, including speech recognition [18], data compression [19], image processing [20], fault diagnosis [21], and signal denoising [22]. As a powerful signal processing technique, SVD exhibits excellent performance in mechanical fault diagnosis. Unlike the traditional decomposition algorithm, SVD ensures the stability of feature extraction based on the theory of matrix transformation [23]. For monitoring the condition of rotating machinery, Yang and Tse developed a denoising method of vibration signals by singular entropy; it studied the distribution characteristics of the noise and clean signals [24]. In addition, Golafshan and Sanliturk developed a novel SVD-based denoising method, which was successfully applied for ball bearing localized fault detection in both the time and frequency domains of the vibration signals [25].

However, there are two critical problems in SVD signal denoising: the selection of the construction matrix and determination of the effective singular values. Initially, a one-dimensional signal must be constructed in the trajectory matrix based on the matrix transformation principle of SVD. The common matrix forms include the Toeplitz matrix, cycle matrix, and Hankel matrix [26], of which the most widely used is the Hankel matrix. In reference [27], it was proven that an original signal could be decomposed into a linear superposition of a series of component signals by SVD using the Hankel matrix. Zhao and Ye pointed out that SVD based on the Hankel matrix was quite similar to the signal processing effect of wavelet transform [11]. In 2015, Jiang et al. used the singular values of Hankel–SVD as the characteristic parameters to diagnose bearings [28]. For the order determination of singular values, energy-based methods can appropriately select the active order under the premise of good prior knowledge, such as entropy increments [24] and cumulative contributions of the singular values [29]. In 2010, Zhao et al. used a curvature spectrum of singular values to choose the order of the valid singular values, thus reliably determining the total number of bearing raceway peeling pits [30]. Furthermore, numerous studies have been devoted to the analysis of the difference spectrum relying on the abrupt change of singular values to reduce noise [31]. Li et al. found a unique relationship between valid singular values and major frequencies, which assisted in the inverse verification of the singular value order [32]. In 2016, Zhang et al. completed order determination based on the difference of singular value variance, and thus extended SVD to the denoising of non-periodic signals [33]. When dealing with complex vibration signals in SVD-based denoising, the accuracy and robustness of the order determination are still the most significant properties.

To reduce noise effectively, a signal denoising method based on SVD and the Akaike information criterion (AIC) is proposed. This method can solve the problems of the selection of matrix structure and order determination of singular values. Based on the energy characteristics of the singular values, the optimal structure of the Hankel matrix is determined to act as the trajectory matrix of the signals. In the process of SVD, the effective singular values are accurately selected by adopting the improved AIC. After eliminating noise components, the remaining singular components are used to reconstruct an approximate matrix. Finally, the averaging method is utilized to obtain the denoising time series signal.

The remainder of this paper is organized as follows. Section 2 briefly reviews the principles of the SVD and AIC. Section 3 describes AIC–SVD to make it applicable for vibration signals containing colored noise. The effectiveness of the proposed method is verified by simulation analysis, as presented in Section 4, and the application of a reaction wheel, as described in Section 5. Finally, in Section 6, the conclusions are drawn.

## 2. Theoretical Background

### 2.1. Singular Value Decomposition of Signals

SVD is an orthogonal transformation. For a real matrix, $A\in R^{m\times n}$, there exist two orthogonal matrices, $U\in R^{m\times m}$ and $V\in R^{n\times n}$, that satisfy the equation given below [14]

(1) $$A=U\Sigma V^{T}=\sum_{i=1}^{q}u_{i}\sigma_{i}v_{i}{}^{T},$$

where the diagonal matrix, Σ, is $\left[diag(\sigma_{1},\sigma_{2},\cdots ,\sigma_{q}),0\right]$ or its transposition. The elements, $\sigma_{i}$($\sigma_{1}>\sigma_{2}>\cdots >\sigma_{q}$), are the singular values of the matrix A, and q=min(m,n). U and V are the unitary matrices of A, and their column vectors $u_{i}$ and $v_{i}$ are the eigenvectors of the covariance matrices, $AA^{T}$ and $A^{T}A$, respectively. 

The singular values correspond to the feature components of the decomposition matrix. Apart from their high stability, they also have the characteristics of proportional and rotational invariance. Therefore, SVD can ensure the robustness of the signal features represented by different singular values, in compliance with the properties required by the feature vectors in pattern recognition. In the SVD-based process of signals, the Hankel matrix is typically accepted as the trajectory matrix because of its characteristic of zero phase shift [11]. A signal containing a noise is indicated as a vector form, s=[s(1),s(2),⋯,s(N)], and its corresponding *m* × *n* dimensional Hankel matrix form is expressed as

(2) $$A={\left(a_{ij}\right)}_{m\times n}=\left[\begin{array}{cccc} s(1) & s(2) & \cdots & s(n)\\ s(2) & s(3) & \cdots & s(n+1)\\ \vdots & \vdots & & \vdots \\ s(m) & s(m+1) & \cdots & s(N) \end{array}\right],$$

where m=N−n+1 and 1<n<N.

The sampling signal can be expressed by Equation (2) as

(3) s=[A(1,:),A(2:m,n)].

Defining $A_{i}=u_{i}\sigma_{i}v_{i}{}^{T}$, the signal component, $P_{i}$, can be expressed as [28]

(4) $$P_{i}=[A_{i}(1,:),A_{i}(2:m,n)].$$

Based on Equations (1), (3), and (4), the original signal can be written as

(5) $$s=\sum_{i=1}^{q}P_{i}.$$

Based on Equation (5), by SVD using the Hankel matrix, the polluted signal can be decomposed into a simple linear superposition of a series of component signals [27]. For an additive noise signal, $s=x+w_{noise}$, an advantage of this decomposition is that the clean signal can be solved by the order of the effective singular values.

(6) $$x=\sum_{i=1}^{k}P_{i},$$

where x is the clean signal, and k is the order of the effective singular values.

### 2.2. Order Determination of Akaike Information Criterion

The AIC is an estimated measure of the fitting goodness of statistical models [34], and is currently used in the estimation of the source number. The decision functions of the AIC are as follows [35]:

(7) $$AIC(d)=-2N(n-d)\log_{10}\left(L_{d}\right)+2d(2n-d)$$

and

(8) $$L_{d}=\frac{\prod_{i=d+1}^{n}\lambda_{i}{}^{\frac{1}{n-d}}}{\frac{1}{n-d}\sum_{i=d+1}^{n}\lambda_{i}},$$

where $\lambda_{i}=\sigma_{i}{}^{2}$ denotes the eigenvalues of the unitary matrices, $L_{d}$ is the maximum likelihood estimation of the eigenvalues, and d=1,2,⋯,n−1 denotes the number of sources.

The AIC function consists of two parts. The former term is the maximum likelihood estimation of the model parameters, which reflects the parameter fitness of the principal components. The second term is the bias correction term inserted to convert the AIC into an unbiased estimator. The former term decreases with the increase in the number of sources, whereas the second term is contrary to the former. When the sum of the two terms is minimum, the best estimate of the effective order is obtained by balancing both the terms as

(9) $$k=\underset{d}{\arg\min}\left(AIC(d)\right).$$

## 3. Signal Denoising of Akaike Information Criterion–Singular Value Decomposition

### 3.1. Selection of Hankel Matrix Rows and Columns

To select the number of rows and columns of the Hankel matrix, the energy characteristics of the singular values are considered. The energy of the singular values indirectly reflects the information richness of the trajectory matrix [36], which is defined as

(10) $$E(n)=\sum_{i=1}^{q}\sigma_{i}{}^{2}.$$

The relationship between the energy of the singular values and elements of the Hankle matrix can be derived from Equation (11).

(11) $$AA^{T}=[u_{1}u_{2}\cdots u_{q}]\cdot \left[\begin{array}{cccc} \lambda_{1} & & & \\ & \lambda_{2} & & \\ & & \ddots & \\ & & & \lambda_{q} \end{array}\right]\cdot {\left[u_{1}u_{2}\cdots u_{q}\right]}^{T},$$

(12) $$E(n)=\lambda_{1}+\lambda_{2}+\cdots +\lambda_{q}=\sum_{j=1}^{n}\sum_{i=1}^{m}a_{ij}{}^{2}.$$

The difference in the number of rows and columns will modify the singular value energy. To easily distinguish the singular components and avoid feature coupling, the optimal number of matrix columns is selected based on the maximum energy of the singular values, i.e.,

(13) $$\hat{n}=\underset{n}{=\arg\max}\left(E(n)\right)\underset{n}{=\arg\max}\left(\sum_{j=1}^{n}\sum_{i=1}^{N-n+1}a_{ij}{}^{2}\right).$$

According to Equation (13), the energy of the singular values is equal to the sum of the squares of all the matrix elements. When the structure of the Hankel matrix is a square or an approximate square, the corresponding energy of the singular values is maximum. Specifically, if N is even, the energy of the singular values is maximum at n=N/2 and m=N/2+1. If N is odd, the energy of the singular values is maximum at n=m=(N+1)/2. As the basis for selecting the optimal structure of Hankel matrix, the maximum criterion of singular value energy makes it convenient to identify the effective singular components.

### 3.2. Verification and Improvement of Order Determination

To verify the validity of the order determination based on the AIC, the different types of signals are designed. The expressions of the periodic, attenuation and sweep signal are given as

(14) $$\left\{ \begin{array}{l} x_{1}=\sin(40\pi t)+1.8\sin(100\pi t)+0.5\sin(200\pi t)\\ x_{2}=\exp^{-2t}[\sin(40\pi t)+0.5\sin(200\pi t)]\\ x_{3}=chirp(t,10,1,100) \end{array}\right..$$

**Figure 1:**
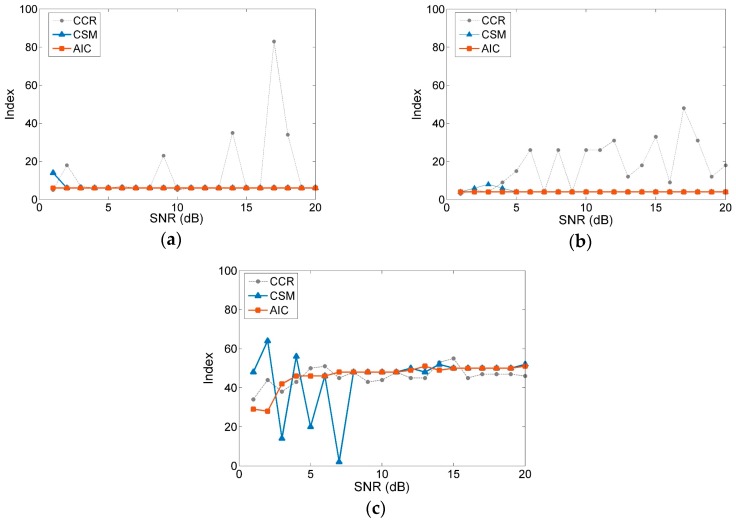
Comparison of the different methods in order determination: (**a**) signals $s_{1(SNRs)}$; (**b**) signals $s_{2(SNRs)}$; (**c**) signals $s_{3(SNRs)}$. CCR: cumulative contribution rate; CSM: singular value curvature spectrum; AIC: Akaike information criterion; SNR: signal-to-noise ratio.

Mixed with Gaussian white noise of different signal-to-noise ratios (SNRs), the initial signals turn into a series of polluted signals $s_{1(SNRs)}$, $s_{2(SNRs)}$ and $s_{3(SNRs)}$, respectively. At a sampling rate of 1 kHz and sampling time of 1 s, the polluted signals are constructed as 501 × 500 Hankel matrices to calculate by SVD. For simulation signals of different SNRs, the AIC is used to determine orders in comparison with cumulative contribution rate (CCR) and singular value curvature spectrum (CSM), as shown in Figure 1.

Based on the main frequency analysis method, the effective orders of $s_{1(SNRs)}$ and $s_{2(SNRs)}$ can be rapidly determined as 6 and 4. In Figure 1a,b, the results calculated by the AIC are consistent with those by main frequency analysis method, remaining constant irrespective of the change in the SNR. Concurrently, violent jumps occur in the curves of both the CCR and CSM. As can be observed in Figure 1c, the effective orders by the AIC are more stable than the compared methods for sweep signals $s_{3(SNRs)}$. Therefore, the AIC improves the accuracy and robustness of the order determination, yielding results better than those obtained with other methods at different SNRs. The AIC can achieve viable noise separation, which is beneficial for reasonable noise reduction and feature extraction.

Apart from a white noise of uniform power, the actual vibration signals are also mixed with an uneven colored noise. To smooth the interference components in the background of the colored noise, the eigenvalues are modified by the diagonal loading technique [37] as follows:

(15) $$\mu_{i}=\sigma_{i}{}^{2}+\sqrt{\sum_{i=1}^{n}\sigma_{i}{}^{2}}.$$

Substituting the modified eigenvalues into the maximum likelihood estimation of the signals, the improved AIC function becomes as expressed in Equation (16). Therefore, the adaptive determination of the singular components can be achieved by minimizing the AIC objective function for the signals containing the colored noise.

(16) $$AIC(d)=-2N(n-d)\log_{10}\left(\frac{\prod_{i=d+1}^{n}\mu_{i}{}^{\frac{1}{n-d}}}{\frac{1}{n-d}\sum_{i=d+1}^{n}\mu_{i}}\right)+2d(2n-d).$$

### 3.3. Denoising of Akaike Information Criterion–Singular Value Decomposition

**Figure 2:**
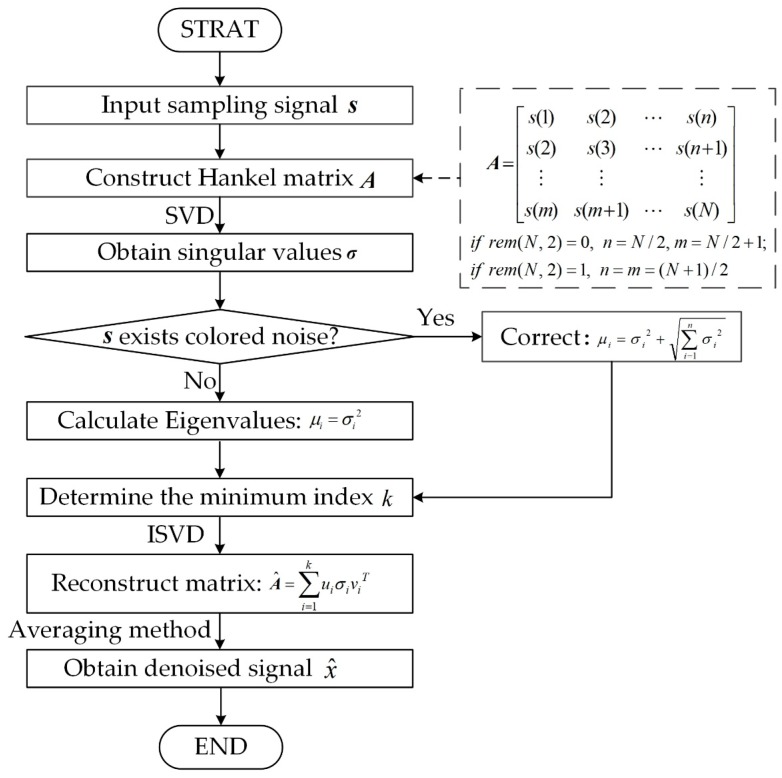
Flow chart of the signal denoising method using AIC–SVD. SVD: singular value.decomposition.

Combining the energy characteristics and AIC-based order determination of the singular values, a signal denoising method called AIC–SVD is proposed, as shown in Figure 2. The detailed steps of the method can be described as follows:

Step 1. An *m* × *n* dimension Hankel matrix is chosen as the trajectory matrix of the sampling signal, s=[s(1),s(2),⋯,s(N)], and then the optimal number of rows and columns of the matrix is selected according to the maximum energy criterion of the singular values;
Step 2. SVD is performed on the optimal construction matrix to obtain a sequence of non-zero singular values, $\sigma =(\sigma_{1},\sigma_{2},\cdots ,\sigma_{q})$. For signals containing the colored noise, the eigenvalues are corrected according to Equation (16). Next, the index of the minimum AIC value is determined by using the AIC, which is the order of effective singular values;
Step 3. The inverse operation of SVD is applied to the singular components of the forward k order to obtain the approximate matrix, $\hat{A}$;
Step 4. According to the averaging method expressed in Equation (17), the denoised signal is obtained by the reconstruction of the time series signals from the approximate matrix.
  (17) $$\hat{x}(i)=\frac{1}{h-l+1}\sum_{j=l}^{h}\hat{A}(i-j+1,j),$$
  where i=1,2,…,N, l=max(1,i−n+1), and h=min(n,i).

## 4. Simulation of Akaike Information Criterion–Singular Value Decomposition

### 4.1. Numerical Simulation

**Figure 3:**
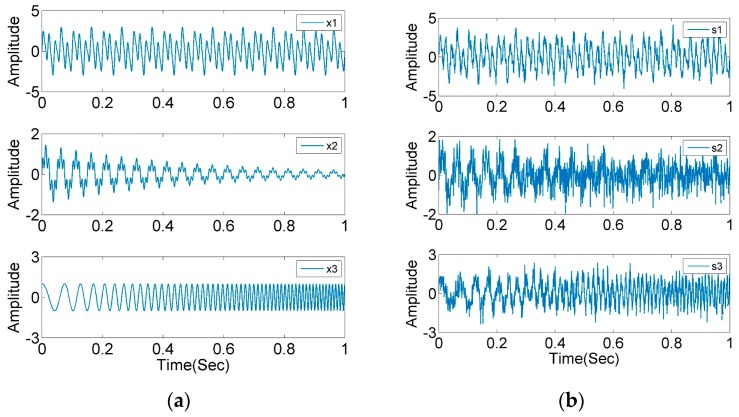
Waveform diagrams of simulation signals: (**a**) clean signals; (**b**) polluted signals with Gaussian white noise of 5 dB.

**Figure 4:**
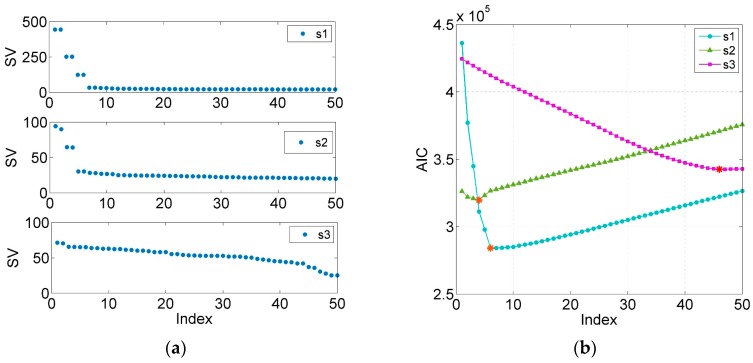
Calculation of simulation signals based on AIC–SVD: (**a**) singular values; (**b**) AIC values.

To verify the effectiveness of AIC–SVD in signal denoising, simulation experiments are performed with signal $s_{1}$, $s_{2}$ and $s_{3}$ mixed with a Gaussian white noise of 5 dB. At a sampling rate of 1 kHz and sampling time of 1 s, the corresponding waveform diagrams of the clean and polluted signals are shown in Figure 3. After selecting 501 × 500 Hankel matrices to construct the trajectory matrix of the signals, the singular values and the AIC values are calculated, as shown in Figure 4. Concurrently, the relevant parameters are extracted and listed in Table 1.

**Figure 5:**
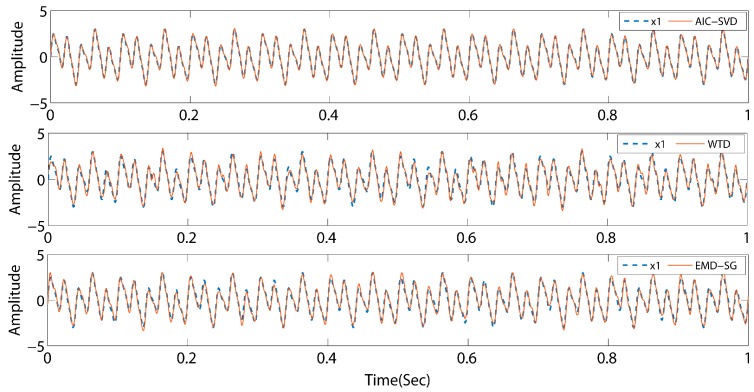
Comparison of denoising effects by different methods for signal $s_{1}$. WTD: wavelet threshold denoising; EMD-SG: empirical mode decomposition with Savitzky–Golay filter.

**Figure 6:**
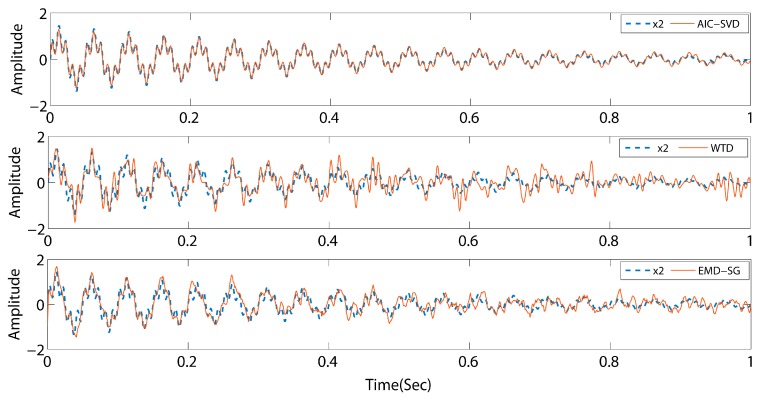
Comparison of denoising effects by different methods for signal $s_{2}$.

**Figure 7:**
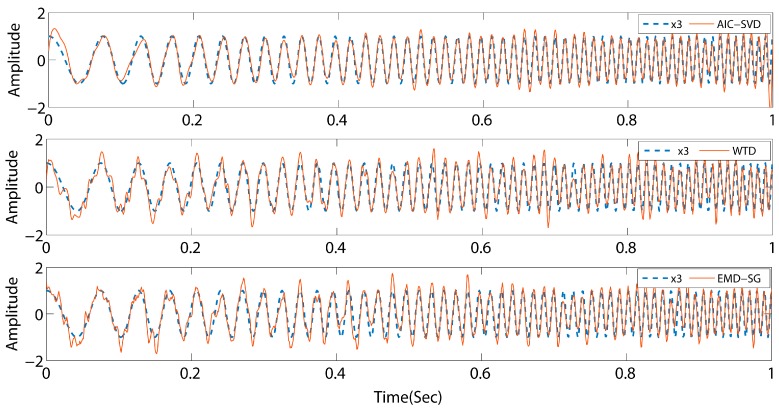
Comparison of denoising effects by different methods for signal $s_{3}$.

As listed in Table 1, the minimum AIC value indices of the above-mentioned three signals are 6, 4, and 46, respectively. Concurrently, the corresponding effective singular spectral values are 89.19%, 54.82%, and 66.75%. The values of the valid singular spectrum are extremely close to the energy ratio of the initial pure signals, and the maximum error is 8.36%. This illustrates that the AIC exhibits a high performance for the order determination of singular values. To determine the reliability of the method, AIC–SVD is compared to WTD and EMD–SG by reconstructing the signals. The processed signals are shown in Figure 5, Figure 6 and Figure 7.

The comparison reveals that the signals processed by AIC–SVD are well restored by the morphology of the pure signal without a phase shift. For a periodic signal, the denoising effects of WTD and EMD–SG are similar overall to that of AIC–SVD. However, the attenuated signal and swept frequency signal have a notable issue. Specifically, the reconstructed signals exhibit a major waveform distortion, which is not conducive to the subsequent extraction and analysis of the features. The denoising method of AIC–SVD can prevent signal distortion while effectively removing noise. With zero phase shift characteristics, the method of AIC–SVD is suitable in the denoising of different types of signals.

### 4.2. Denoising Performance Evaluation

To describe the performance of denoising more intuitively and accurately, the simulation signals are further quantitatively analyzed by combining the SNR, root mean square error (RMSE), and waveform correlation coefficient (NCC). These evaluation indicators are defined as follows [38]:

(18) $$SNR=10\log_{10}\frac{\sum_{i=1}^{N}x{\left(i\right)}^{2}}{\sum_{i=1}^{N}{\left[x(i)-\hat{x}(i)\right]}^{2}},$$

(19) $$RMSE=\sqrt{\frac{1}{N}\sum_{i=1}^{N}{\left[x(i)-\hat{x}(i)\right]}^{2}},$$

(20) $$NCC=\frac{\sum_{i=1}^{N}x(i)\cdot \hat{x}(i)}{\sqrt{\left(\sum_{i=1}^{N}x{\left(i\right)}^{2}\right)\cdot \left(\sum_{i=1}^{N}\hat{x}{\left(i\right)}^{2}\right)}}.$$

The SNR and RMSE reflect the global characteristics of the denoising performance, whereas the NCC describes the local characteristics of the signals. To avoid the limitations of a single evaluation index, a comprehensive evaluation index (CID) of denoising is introduced by integrating the SNR, RMSE and NCC. It can be defined as

(21) $$CID=\frac{SNR\cdot NCC}{RMSE}.$$

**Figure 8:**
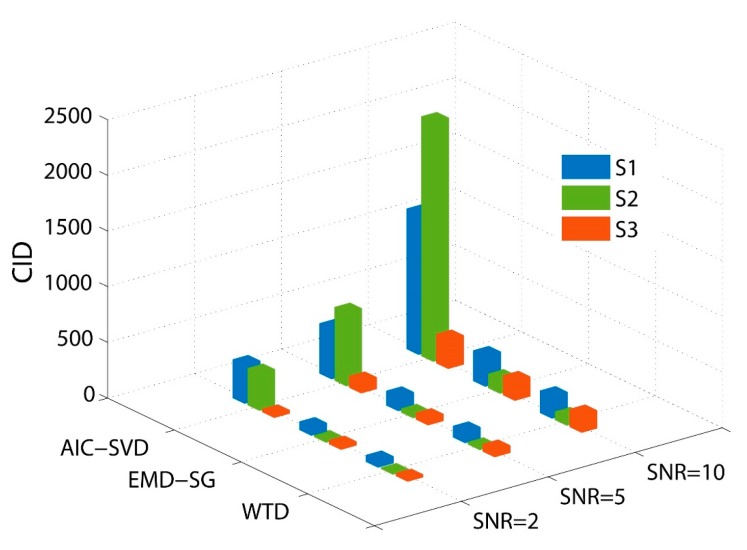
Comparison of the CID for the different denoising methods.

According to Equation (21), a large value of CID corresponds to a good performance in signal denoising. For the simulation signals, the denoising performance parameters of different methods are calculated and listed in Table 2. Subsequently, Gaussian white noise with different SNRs (2 dB, 5 dB, and 10 dB) is added to the pure signals. The CID values of the denoising at the different SNRs are shown in Figure 8.

The data in Table 2 prove that the SNRs of the signals are improved after denoising by both the methods, of which AIC–SVD leads to the largest increase. The minimum NCC value of AIC–SVD is 0.954, which can preserve the local waveform characteristics of the initial signal well, avoiding signal distortion. In Figure 8, the CID values of the different denoising methods increase with the improvement in the SNR of the initial signal, and the overall denoising performance of AIC–SVD is significantly better than those of the compared methods. Specifically, for the attenuated signal, the corresponding CID value of AIC–SVD at a 5 dB SNR is 657, which is much larger than those of the other methods. The powerful denoising performance of AIC–SVD for the attenuated signal shows that it is an effective pre-processing tool for vibration signals with pulse characteristics.

## 5. Study on Micro-Vibration Signal Denoising of Reaction Wheels

### 5.1. Micro-Vibration Test

**Figure 9:**
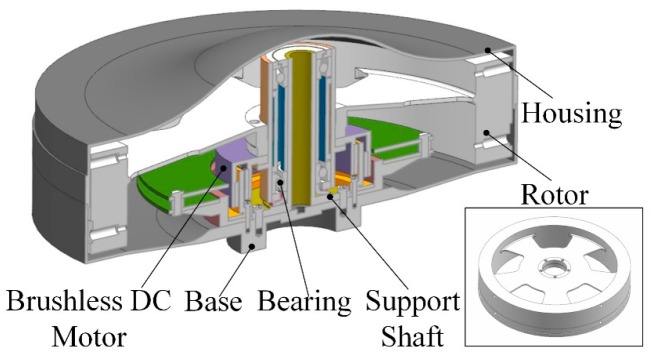
Structure of a reaction wheel. DC: direct current.

As important attitude control components of a satellite, reaction wheels have the general characteristics of rotating machinery. The specific structure of a reaction wheel is depicted in Figure 9. It primarily consists of a rotor supported by ball bearings encased in a housing and driven by a brushless direct current (DC) motor. Influenced by some factors such as the internal rotor imbalance, bearing imperfections, and structural modes, a reaction wheel generates disturbance forces and moments during running. The negative impact of the disturbances is unacceptable for the normal operation of the payloads in satellites [39]. To ensure the successful implementation of the operations in space, it is necessary to analyze the micro-vibration characteristics of reaction wheels.

**Figure 10:**
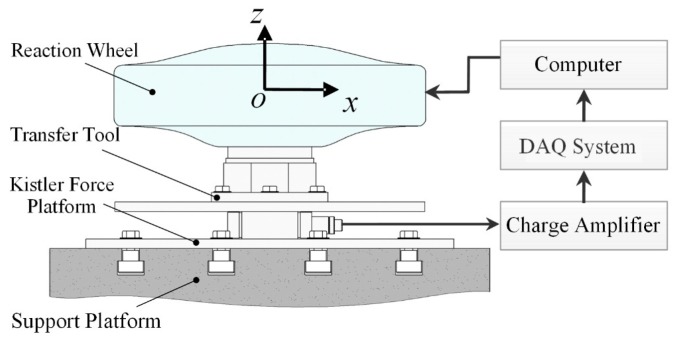
Micro-vibration test of a reaction wheel. DAQ: data acquisition.

An on-ground micro-vibration test is frequently performed as an approach to study the micro-vibration characteristics of reaction wheels. It is conducted using the Kistler micro-vibration test device, as depicted in Figure 10. During the operation of a reaction wheel, the disturbance response is transmitted to the force measurement platform through the transfer tool. Then, the micro-vibration signals collected by piezoelectric sensors are transmitted to a data acquisition (DAQ) system via a charge amplifier, which are displayed and processed on a computer.

**Figure 11:**
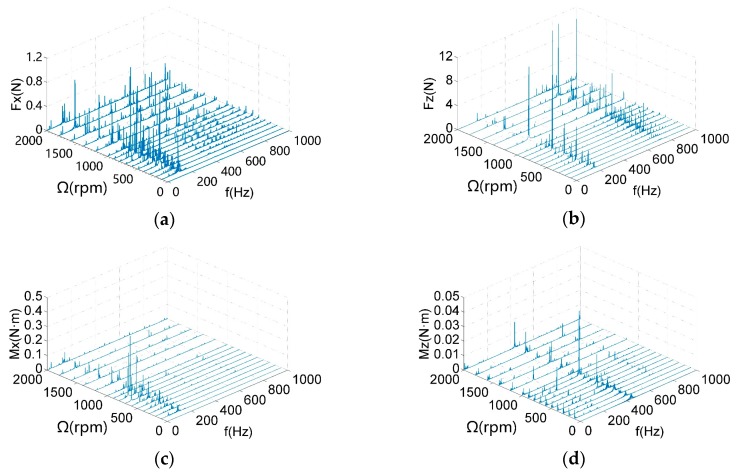
Waterfall diagrams of a reaction wheel: (**a**) $F_{x}$; (**b**) $F_{z}$; (**c**) $M_{x}$; (**d**) $M_{z}$.

The micro-vibration signals of the reaction wheel are collected at different rotational speeds (0–2000 rpm). Performing the fast Fourier transform on the time domain signals, three-dimensional waterfall diagrams of the radial and axial disturbances are obtained, as shown in Figure 11. The vibration of the reaction wheel mainly concentrates on the radial disturbance forces $F_{x}$, axial disturbance forces $F_{z}$, and radial disturbance torque $M_{x}$. Relatively, the magnitude of the axial disturbing moment $M_{z}$ is small, which can be ignored. Therefore, the analysis of the micro-vibration signals is carried out in $F_{x}$, $F_{z}$ and $M_{x}$.

### 5.2. Analysis of Micro-Vibration Denoising

Excluding the environmental factors, the noise of micro-vibration signals is also derived from the internal torque fluctuations and frictional interference. To separate the noise component from the micro-vibration signals, a general processing method called peak threshold denoising is used in reaction wheels at present. Based on the amplitude statistical characteristics of the noise, the threshold value to remove the noise from the original signal is determined. It is described as [40]

(22) $$DT=\mu +N_{\delta }\cdot \delta ,$$

where μ and δ are the mean and standard deviation of the spike amplitude, respectively, and $N_{\delta }$ is a user-defined tolerance level, which also depends on the SNR of the sampling signals. Generally, the value of $N_{\delta }$ can be 2 or 3.

**Figure 12:**
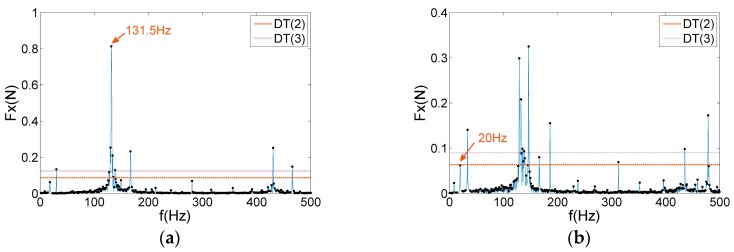
Peak threshold denoising of the micro-vibration signals: (**a**) 1800 rpm; (**b**) 2000 rpm.

**Figure 13:**
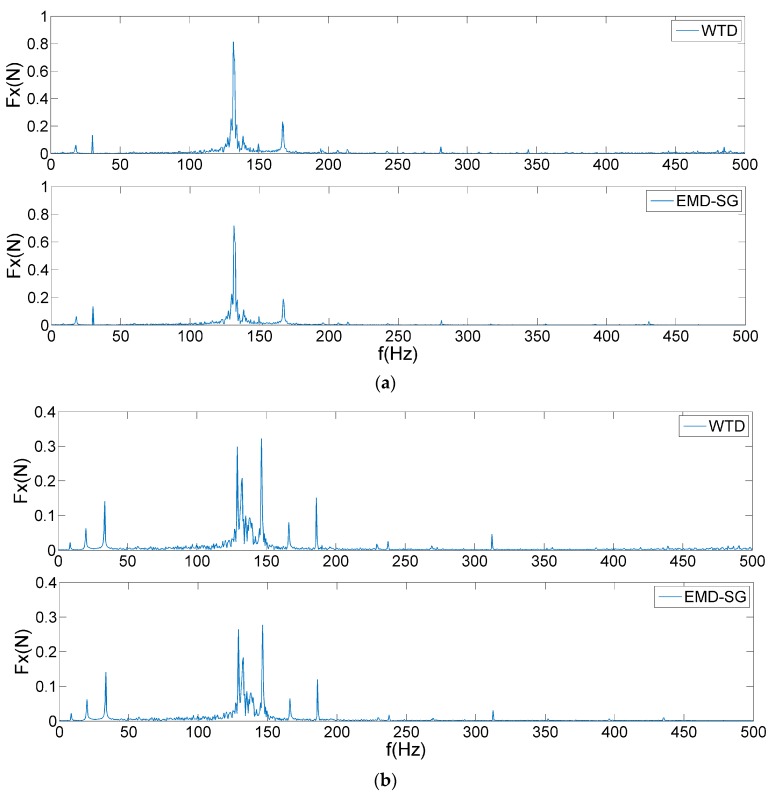
Spectra of micro-vibration signals by WTD and EMD–SG: (**a**) 1800 rpm; (**b**) 2000 rpm.

In the study of a reaction wheel under the ultimate working conditions, the micro-vibration signals at 1800 and 2000 rpm are selected for the denoising analysis. The frequency of interest is set within 500 Hz, which is the main frequency band that causes satellite jitter. The threshold values are calculated according to different tolerance levels, as shown in Figure 12. Similarly, WTD and EMD–SG are used to suppress noise in micro-vibration signals, as shown in Figure 13.

As exhibited in Figure 12, the magnitude of the user tolerance level directly affects the final effect of the signal denoising. In Figure 12a, a reaction wheel generates a large disturbance force at 131.5 Hz owing to the coupling of the harmonic responses and structural modes, which increases the threshold value to filter out some critical frequency features. Some obvious feature frequencies are equally easy to be removed in Figure 12b, such as 20 Hz. As shown in Figure 13, WTD and EMD–SG mainly act on high-frequency of test signals, which appear under-denoising in the low-frequency range and lose super-harmonics. The filtered details frequently indicate that the system experiences a significant motion mechanism, which is not conducive to the subsequent characteristic analysis and fault diagnosis.

**Figure 14:**
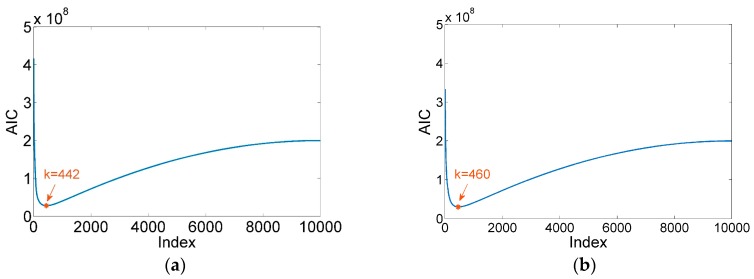
AIC diagrams at different rotational speeds: (**a**) 1800 rpm; (**b**) 2000 rpm.

**Figure 15:**
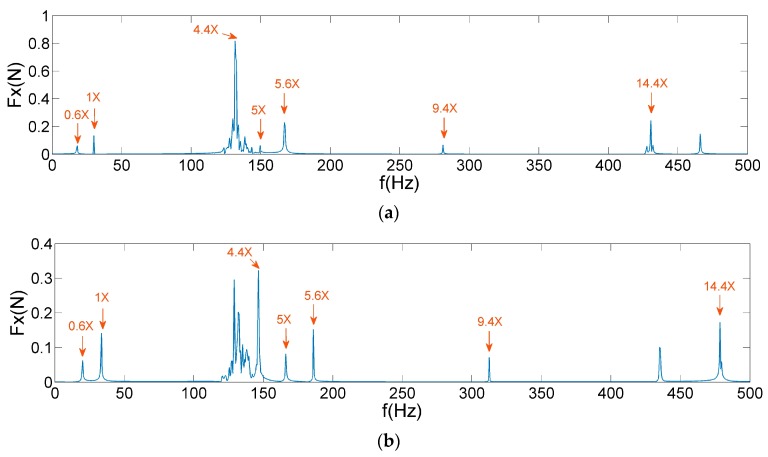
AIC–SVD denoised frequency spectrum of $F_{x}$: (**a**) 1800 rpm; (**b**) 2000 rpm.

Owing to inappropriate parameter setting and resonance coupling, these denoising methods can easily cause phenomena of over-denoising and under-denoising. Therefore, AIC–SVD is introduced into the pre-processing of the micro-vibration signals of the above-mentioned reaction wheel. By constructing the Hankel matrix of micro-vibration signals, the singular values are solved by SVD. Owing to the presence of colored noise in the micro-vibration signals, the improved AIC is used to determine the order of the effective singular value by correcting the eigenvalues. According to the calculation results as shown in Figure 14, the indices of minimum AIC value is selected to reconstruct the approximate matrixes. Once the time series signals are restored by the averaging method, denoised frequency spectra are obtained, as shown in Figure 15.

**Figure 16:**
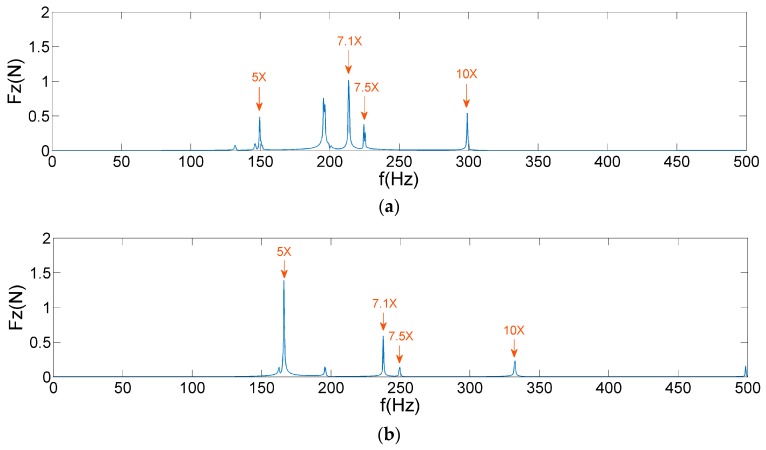
AIC–SVD denoised frequency spectra of $F_{z}$: (**a**) 1800 rpm; (**b**) 2000 rpm.

**Figure 17:**
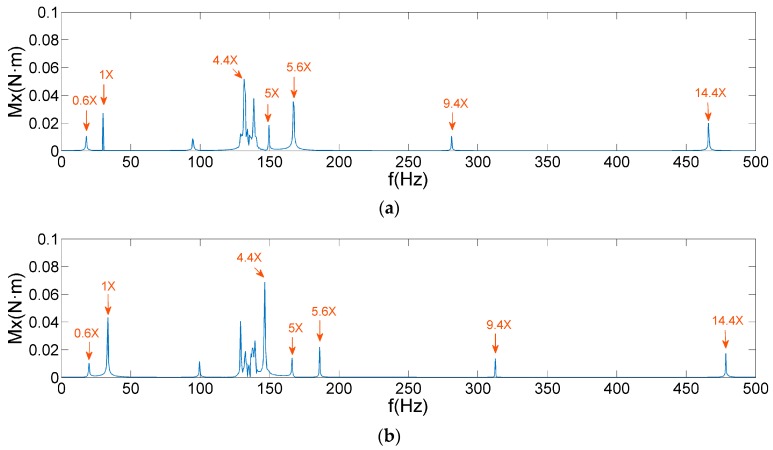
AIC–SVD denoised frequency spectra of $M_{x}$: (**a**) 1800 rpm; (**b**) 2000 rpm.

By comparing Figure 12, Figure 13 and Figure 15, it is observed that AIC–SVD can effectively eliminate the noise from the micro-vibration signals. The denoised signals are convenient in the extraction of harmonic features. As shown in Figure 16 and Figure 17, the micro-vibration signals of $F_{z}$ and $M_{x}$ are processed by AIC–SVD. And the related parameters of the reaction wheel are listed in Table 3.

The data listed in Table 3 provide all the harmonic coefficients and related frequencies. The average running time of AIC–SVD is 443 s, which is mainly caused by SVD of matrices at the high sampling frequency. Combined with the analysis of the disturbance mechanism, it reveals that the denoised signals include a fundamental harmonic caused by the rotor imbalance, a sub-harmonic of 0.6 times frequency caused by the bearing cage defects, and super-harmonics. Super-harmonics contain 4.4, 5, 5.6, 9.4, and 14.4 times frequency in both $F_{x}$ and $M_{x}$, 5, 7.1, 7.5, and 10 times frequency in $F_{z}$, which are caused by the coupling of bearing imperfections.

## 6. Conclusions

This paper presents a powerful denoising method based on SVD and the improved AIC. Simulation analysis and an engineering application are undertaken to demonstrate the effectiveness of the proposed AIC–SVD, and the following conclusions can be drawn:

(1) In the signal processing of SVD based on Hankel matrix, the energy of the singular values is maximum when the matrix structure is a square or an approximate square. Currently, the feature components provide the largest degree of distinction, which is convenient for the order determination of the effective singular values.
(2) The method of order determination based on the AIC possesses high accuracy and robustness. Furthermore, AIC–SVD is significantly better than WTD and EMD–SG in the denoising performance for the signals containing Gaussian white noise.
(3) In the micro-vibration signal pre-processing of reaction wheels, AIC–SVD achieves a reasonable denoising effect for the signals containing Gaussian white noise and colored noise. This solves the problem of over-denoising and under-denoising caused by inappropriate parameter selection and modal resonance factor. The proposed method has strong adaptability to vibration signal processing under different working conditions, which is beneficial in the extraction of harmonic features.

By extracting the harmonic parameters, a reasonable disturbance model of reaction wheels will be established to describe the characteristics of micro-vibration. This will be of far-reaching significance in the study of orbital-operation monitoring and vibration-reduction space satellites. In addition, there is still room for improvement in the running efficiency of AIC–SVD, which would be optimized in the following study.

## Figures and Tables

**Table 1:** Simulation parameters of signals based on AIC–SVD.

Signal	*k*	SV	AIC	Energy Ratio ^1^	Valid Singular Spectrum	Error
*s*_1_	6	127.8	2.485 × 10^5^	84.49%	89.19%	5.92%
*s*_2_	4	53.4	2.727 × 10^5^	59.82%	54.82%	8.36%
*s*_3_	46	32.9	3.426 × 10^5^	63.84%	66.75%	4.56%

^1^ Energy ratio is the energy ratio of clean signals to polluted signals.

**Table 2:** Denoising performance parameters at SNR of 5 dB.

Evaluation Parameters	WTD			EMD–SG			AIC–SVD		
	*s*_1_	*s*_2_	*s*_3_	*s*_1_	*s*_2_	*s*_3_	*s*_1_	*s*_2_	*s*_3_
SNR	31.548	7.196	19.007	34.548	9.908	18.334	51.407	38.295	23.496
RMSE	0.309	0.273	0.274	0.266	0.239	0.283	0.115	0.058	0.219
NCC	0.979	0.800	0.933	0.985	0.845	0.933	0.997	0.990	0.954
CID	100	21	65	128	35	60	447	657	103
Computing time (s)	0.9	1.2	1.4	3.6	2.5	2.4	3.7	2.7	2.7

RMSE: root mean square error; NCC: waveform correlation coefficient; CID: comprehensive evaluation index.

**Table 3:** Characteristic parameters of micro-vibration signals by AIC–SVD.

Disturbing Component	Speed (rpm)	k	Valid Singular Spectrum	Computing Time (s)	Harmonic Coefficient
*F_x_*	1800	442	94.9%	436	0.6, 1, 4.4, 5, 5.6, 9.4, 14.4
	2000	460	92.4%	448	
*F_z_*	1800	422	99.3%	444	5, 7.1, 7.5, 10
	2000	498	98.9%	450	
*M_x_*	1800	64	86.7%	440	0.6, 1, 4.4, 5, 5.6, 9.4, 14.4
	2000	68	88.1%	442	
